# Launching the Global Health Network Middle East and North Africa Regional Network: A Path to Promote the Region’s Global Health Research Presence and Build Unity and Collaboration Towards Tackling Regional Public Health Priorities

**DOI:** 10.3390/healthcare13121360

**Published:** 2025-06-06

**Authors:** Malak Alrubaie, Rode Amsal Tarekegne, Sania Rahman, Parinita Manikandan, Salvia Zeeshan, Marina AlBada, Trudie Lang, Aseel A. Takshe, Mohammed Alkhaldi

**Affiliations:** 1 The Global Health Network-The Middle East and North Africa Network (TGHN-MENA), Dubai P.O. Box 117781, United Arab Emirates; malakrubi@gmail.com (M.A.); 20230004137@students.cud.ac.ae (R.A.T.); 20230004332@students.cud.ac.ae (S.R.); parinita.manikandan@gmail.com (P.M.); salvia.zeeshan@ndm.ox.ac.uk (S.Z.); marina.albada@ndm.ox.ac.uk (M.A.); 2 Faculty of Health, University of Waterloo, Waterloo, ON N2L 3G1, Canada; 3 School of Health Sciences and Psychology, Department of Public Health, Canadian University Dubai, Dubai P.O. Box 117781, United Arab Emirates; aseel.takshe@cud.ac.ae; 4 Centre for Tropical Medicine and Global Health, Nuffield Department of Medicine, University of Oxford, Oxford OX1 2JD, UK; trudie.lang@ndm.ox.ac.uk; 5 School of Physical and Occupational Therapy, Faculty of Medicine and Health Sciences, McGill University, Montreal, QC H3A 0G4, Canada

**Keywords:** Global Health Network, MENA region, public health education, health-related SDGs, health equity, capacity building

## Abstract

The Global Health Network Middle East and North Africa (TGHN MENA) was officially launched on 21 October 2024, representing a pivotal initiative to address the region’s distinct and complex public health challenges. Building on the comprehensive global framework of the central TGHN network, the regional TGHN MENA network was founded by region-based experts with support from the TGHN team. The network was established as a pioneering initiative to bring together 18 partners from 14 countries, representing various sectors such as academia, policymakers, and governmental and non-governmental organizations, to tackle pressing issues such as chronic diseases, mental health, and climate change impacts. High-level panel discussions were held to define the goals of TGHN MENA in building resilient public health systems. This perspective outlines the network’s vision for building resilient health systems through research prioritization and capacity strengthening, amidst growing uncertainties in the regional public health landscape. The MENA region has diverse and complex public health challenges related to health systems, emergencies, chronic disease, mental health disorders, and climate change, due to cultural, social, and geographic differences. The TGHN MENA network is a community of practice and can identify commonalities and priorities and find shareable solutions. Key strategies proposed include establishing an open-access, online platform to support knowledge exchange, implementing on-the-job training and capacity-strengthening initiatives, and emphasizing the use of artificial intelligence in public health research. This perspective outlines TGHN MENA’s inaugural one-year action plan, which emphasizes regular knowledge-sharing activities, capacity-building initiatives, and sustained partners’ commitment as foundational steps towards improved public health outcomes in the region.

## Summary Points

Launched in October 2024, the TGHN MENA network unites 18 partners from 14 countries to address unique public health challenges in the Middle East and North Africa region through collaborative research and capacity building; The network’s seven strategic objectives aim to enhance stakeholder collaboration, address regional needs, and strengthen the health research capacity; Inaugural one-year action plan focuses on knowledge-sharing activities, capacity-building initiatives, and establishing connections with global institutions to improve health outcomes in marginalized settings; The successes of the TGHN MENA network will depend on navigating regional complexities, promoting sustained collaboration, and translating research findings into tangible health outcomes.

* * 

The rapidly evolving health challenges of the 21st century demand agile, robust, and coordinated responses. The Global Health Network (TGHN) has consistently been delivering longstanding and impactful efforts to address pressing health issues among different global regions, facilitating the transfer of knowledge and expertise [1,2]. Ongoing disease burdens, frequent outbreaks, health system inefficiencies, and the world’s most severe humanitarian and climate change crises underscore the necessity of collaborative efforts within such dynamic and equitable collaborative networks to address the most pressing and dynamic health challenges faced globally [3,4]. This requires nations and entities from the Global South and Global North to identify key health priorities and mobilize resources effectively to achieve global health equity. Research, as evidenced by the COVID-19 pandemic, remains the most effective pathway to resolution and preparedness [5]. The pandemic demonstrated that robust research efforts are essential in identifying solutions, guiding public health responses, and building resilience against future health emergencies [5]. TGHN bridges the gap between stakeholders and implementers, ensuring inclusive and impactful initiatives for equitable health outcomes. For over a decade, it has united diverse stakeholders, facilitating knowledge exchange and best practices. Its primary goal is to mobilize knowledge, foster collaboration, and generate evidence to improve health outcomes in marginalized settings and disease areas.

The World Health Organization (WHO) underscores the importance of strategic partnerships as a cornerstone of its initiatives, recognizing that addressing the complexities of global health challenges requires integrated, people-centered approaches and collaborative efforts across multiple sectors to strengthen health systems [6]. These collaborative efforts are vital in achieving the Sustainable Development Goals (SDGs), particularly those related to “Goal 3: Ensure healthy lives and promote well-being for all at all ages”, which the network addresses through initiatives aimed at strengthening health systems and improving access to quality healthcare; “Goal 10: Reduce inequality within and among countries”, which TGHN MENA addresses through efforts to reduce health disparities and promote equity in healthcare access; and “Goal 17: Strengthen the means of implementation and revitalize the global partnership for sustainable development”, which the network supports through its cross-sector collaborations and resource-sharing frameworks [7]. The WHO appointed TGHN as a collaborating center in 2022, acknowledging its commitment to enabling research in every healthcare setting within the most underserved regions. Furthermore, the pursuit of Universal Health Coverage (UHC), which aims to ensure that all individuals have access to essential health services without suffering financial hardship, further underscores the importance of these collective, pragmatic endeavors and aligns with the regional priorities in the Eastern Mediterranean, where efforts focus on strengthening health systems, improving equity, and expanding access to quality care for vulnerable populations across the MENA region [8]. By pooling resources and expertise, TGHN identifies and addresses the systemic barriers to research that perpetuate health inequities, thus advancing health justice [9]. In this context, the enduring efforts of TGHN are beneficial and essential in fostering a healthier, more equitable world, making its alignment with common goals indispensable in today’s public health landscape [10].

The Middle East and North Africa (MENA) region is confronted with a unique set of challenges, including perpetual humanitarian crises across multiple countries, which further exacerbate the existing health burdens [11]. These challenges complicate the region’s ability to address a wide range of challenges, including chronic diseases, mental health disorders, health workforce shortages, health system provision and emergency preparedness inadequacies, and adverse climate change impacts [11]. These complexities highlight the urgent need for a truly collaborative approach within the region to tackle these challenges effectively [12]. At the governance and authority level, gaps in health research prioritization hinder meaningful evidence generation and translation to improve local practices [12]. Despite the growing commitment to public health research and policy development, persistent health system-related challenges such as ongoing armed conflicts, mass displacement, fragile health infrastructures, and disparities in healthcare access remain, in addition to health research-related challenges such as limited sustainable resources and capacities and research prioritization and translation [13,14,15,16]. These issues are considered priorities for TGHN and its partners, who are collectively adapting actionable plans—including targeted research on health system strengthening, workforce development, and adaptation to climate-related health threats.

**Figure 1:**
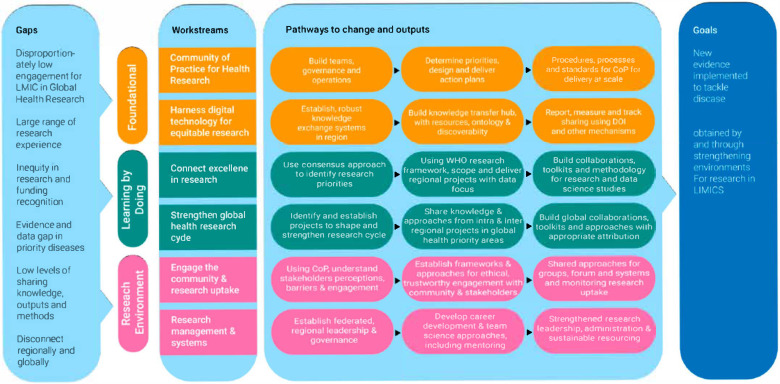
Theory of change adopted by TGHN MENA network.

As shown in Figure 1, the TGHN MENA network’s plan adopted the theory of change framework to collaborate with regional partners in addressing the common challenges and gaps across countries, health systems, public health areas, and partnering institutions. The TGHN MENA network strengthens health research by enhancing core research functions, fostering continuous learning, and empowering the regional research environment [17]. Through collaboration, capacity building, and resource sharing, it addresses regional health priorities, integrates policy and knowledge, and supports sustainable partnerships to achieve impactful public health outcomes.

**Figure 2:**
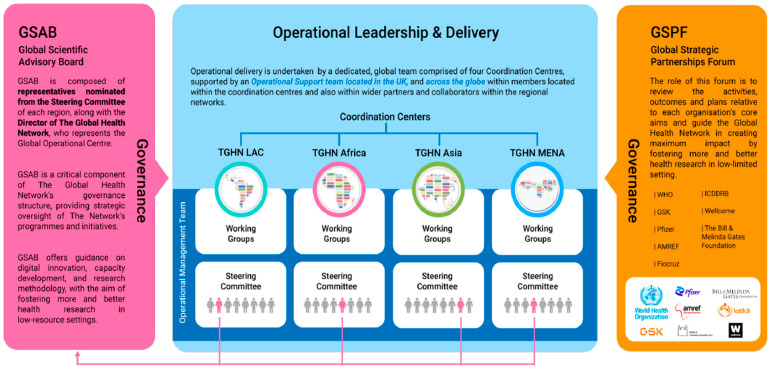
The Global Central TGHN structure.

The Global Central TGHN structure is built around a central operations team, a Global Scientific Advisory Board, and a Strategic Partners Forum, all of which provide strategic direction and governance for the network. As illustrated in Figure 2, this structure is complemented by regional coordination centers and steering committees, enabling seamless data flow, collaboration, and knowledge sharing between global and regional levels while maintaining a unified vision and operational excellence across all regions [17].

Building on the comprehensive global framework of the central TGHN network, the regional TGHN MENA network was founded by region-based experts under the leadership of Scientific Lead and Network Coordinator Dr. Mohammed Alkhaldi, with support from the TGHN team. The network was established as a pioneering initiative to serve as a collaborative platform, bringing together stakeholders from academia, governments, and NGOs to coordinate research and implement evidence-based interventions addressing complex regional challenges. It complements existing public health entities to foster unity and synergy on common priorities. Its core strategy involves building a robust Community of Practice, connecting diverse healthcare stakeholders to improve health systems’ performance and outcomes. Seven strategic objectives of the MENA network are identified: enhance collaboration and integration among stakeholders; identify and address the real needs of the region through joint efforts; identify and tackle unique challenges in health policy, research, medical education, and clinical practice; strengthen capacity building in health research; build partnerships with international organizations; conduct and facilitate learning and experiential activities with all partners; and support resource allocation within the region.

The network ensures equal and joint ownership for all partners through its regional advisory board and technical work group. The network currently comprises 18 key partners from 14 countries, representing academia, policymakers, public health ministries, non-governmental and private organizations, and international collaborators. Notable partners include the University of Oxford; the Canadian University Dubai (CUD); Mohammed V University; Institute Pasteur de Tunis; the WHO Eastern Mediterranean Regional Office (EMRO); Zewail City for Sciences and Technology; the University of Gezira; the American University of Beirut (AUB); the Islamic University of Gaza; the Eastern Mediterranean Public Health Network (EMPHNET); the Ministry of Health of Kuwait; the Yemen Ministry of Public Health and Population; the University of Doha for Science and Technology (UDST); and Pakistan Shaukat Khanum Memorial Hospital and Research Center. International collaborators such as McGill University and the Swiss Tropical and Public Health Institute are also key partners. This comprehensive collaborative partnership continues to grow, with new partners and members expected to join in the near future. It is designed to maximize expertise, resource sharing, and regional impacts.

The TGHN MENA network’s inauguration ceremony took place on 21 October 2024, at the CUD, United Arab Emirates, with both in-person and virtual participation. The event marked the beginning of a collaborative effort to address regional public health challenges. Three panel discussions engaged diverse stakeholders in critical conversations about public health in the MENA region.

The first panel discussion, featuring experts from the WHO, McGill University, the Centers for Disease Control and Prevention (CDC) MENA, AUB, and EMPHNET, examined the current and future state of public health in the MENA region. Panelists highlighted disparities in health infrastructure across countries, with some facing significant challenges due to political conflicts and socioeconomic status. The COVID-19 pandemic exposed gaps in the public health workforce, including unclear job descriptions for professionals, creating confusion in educational programs and career roles. Experts emphasized the need for transformative solutions looking ahead, advocating for specialized research strategies, interdisciplinary collaboration, and capacity building. To establish clearer professional identities, they also proposed introducing residency training programs for public health students within ministries and communities, aiming to solidify the importance of public health professionals in healthcare.

The second panel discussion featured findings from the comprehensive scoping review on public health priorities in the MENA region, titled “Mapping Priorities of Public Health Research and Practice in the MENA Region: A Scoping Review”. This study revealed a significant increase in research outputs during the COVID-19 pandemic. Key priorities include strengthening health systems, workforce development, and non-communicable disease prevention. The study emphasized cost-effective measures for managing non-communicable diseases, improving healthcare delivery, and enhancing public health education through advanced programs and risk communication campaigns. Policy priorities focused on cost-effectiveness in healthcare management and identifying health determinants. Audience participation highlighted the importance of collaborative approaches in addressing these priorities. This review provides essential tools to shape the TGHN MENA network’s strategic vision and operational plan, aligning with its mission to improve health outcomes through collaborative research and practice.

The concluding session of the TGHN MENA network’s launch presented its strategic vision to address regional public health challenges and its approach that emphasizes cross-sector collaboration, resource mobilization, and knowledge exchange. A one-year action plan was introduced, featuring monthly seminars, capacity training for professionals, and establishing connections with global health institutions such as the WHO and the University of Oxford. This plan aims to enhance stakeholder engagement, research capacities, cross-country collaboration, and the translation of research into policy and practice. The importance of involving young researchers and professionals was emphasized to bring forth fresh perspectives, and it introduced the TGHN MENA Knowledge Hub, an online portal to support regional activities, provide access to resources in various public health fields, and keep members updated on network events. Emphasis was placed on measuring the network’s impact through indicators such as the number of collaborative projects, research outputs, stakeholder satisfaction, and policy changes influenced, alongside efforts to build resilient public health systems capable of effectively addressing regional challenges.

In conclusion, there is a critical need in the MENA region for robust, dynamic, and diverse collaborative communities and networks capable of transforming public health education, research, policy, and practice. TGHN MENA has been established to respond to this need by creating an open-access and trilingual community of practice. This network represents a significant step forward in addressing pressing public health challenges in the region through enhanced knowledge sharing and collaboration among healthcare stakeholders. The one-year action plan, emphasizing knowledge-sharing and capacity-building initiatives, provides a robust foundation for achieving these goals. While the success of TGHN MENA depends on navigating the region’s complexities, fostering long-term collaboration, and converting research findings into tangible health outcomes, numerous future challenges must be considered. These include maintaining engagement across diverse partners amidst political and socioeconomic uncertainties, ensuring equitable access to resources and opportunities for all stakeholders, and effectively translating research into actionable policies and practices. The TGHN MENA model has great potential for replication in other regions, particularly in low- and middle-income countries facing similar public health challenges, where TGHN can serve as a blueprint for context-specific, collaborative health initiatives.

By leveraging the MENA countries’ strengths and fostering innovation, particularly in artificial intelligence and digital health technologies, the network aims to advance regional public health outcomes and contribute to global health equity. To evaluate the network’s success, a multi-faceted approach will be pivotal, incorporating quantitative metrics such as research outputs, policy influence, and stakeholder satisfaction, as well as qualitative assessments of the network’s impact on local health systems and communities. The continuous monitoring, adaptation, and refinement of strategies will be crucial to maximizing the long-term impacts and contributions to global health equity. By bridging the gap between research and practice, the network can accelerate the generation of new evidence and implementation of interventions, leading to immediate improvements in population health, potentially serving as a model for other regions in achieving global health goals through collaborative, locally focused approaches.
